# Benznidazole treatment decreases IL-6 levels in *Trypanosoma cruzi*-infected human adipocytes differentiated from adipose tissue-derived stem cells

**DOI:** 10.1590/0074-02760220295

**Published:** 2023-10-23

**Authors:** Leyllane Rafael Moreira, Ana Carla Silva, Cíntia Nascimento da Costa Oliveira, Claudeir Dias da Silva Júnior, Amanda Vasconcelos Nascimento, Kamila Kássia dos Santos Oliveira, Ana Karine de Araújo Soares, Karina Lidianne Alcântara Saraiva, Milena de Paiva Cavalcanti, Virginia Maria Barros de Lorena

**Affiliations:** 1 Universidade Federal de Pernambuco, Programa de Pós-Graduação em Medicina Tropical, Recife, PE, Brasil; 2 Fundação Oswaldo Cruz-Fiocruz, Instituto Aggeu Magalhães, Laboratório de Imunoparasitologia, Recife, PE, Brasil; 3 Fundação Altino Ventura, Recife, PE, Brasil; 4 Fundação Oswaldo Cruz-Fiocruz, Instituto Aggeu Magalhães, Núcleo de Plataformas Tecnológicas, Recife, PE, Brasil; 5 Fundação Oswaldo Cruz-Fiocruz, Instituto Aggeu Magalhães, Departamento de Microbiologia, Recife, PE, Brasil

**Keywords:** adipose tissue, Trypanosoma cruzi, immunomodulation, Benznidazole

## Abstract

**BACKGROUND:**

*Trypanosoma cruzi*, which causes Chagas disease (CD), is a versatile haemoparasite that uses several strategies to evade the host’s immune response, including adipose tissue (AT), used as a reservoir of infection. As it is an effective barrier to parasite evasion, the effectiveness of the drug recommended for treating CD, Benznidazole (BZ), may be questionable.

**OBJECTIVE:**

To this end, we evaluated the parasite load and immunomodulation caused by BZ treatment in the culture of adipocytes differentiated from human adipose tissue-derived stem cells (ADSC) infected with *T. cruzi*.

**METHODS:**

The ADSC were subjected to adipogenic differentiation. We then carried out four cultures in which we infected the differentiated AT with trypomastigote forms of the Y strain of *T. cruzi* and treated them with BZ. After the incubation, the infected AT was subjected to quantitative polymerase chain reaction (qPCR) to quantify the parasite load and transmission electron microscopy (TEM) to verify the infection. The supernatant was collected to measure cytokines, chemokines, and adipokines.

**FINDINGS:**

We found elevated secretion of IL-6, CXCL-10/IP-10, CCL2/MCP-1, CCL5/RANTES, and leptin in infected fat cells. However, treatment with BZ promoted a decrease in IL-6.

**MAIN CONCLUSION:**

Therefore, we believe that BZ has a beneficial role as it reduces inflammation in infected fat cells.

Chagas disease (CD), caused by the haemoflagellate protozoan *Trypanosoma cruzi*, represents a serious public health problem. About 6-7 million people are estimated to be infected worldwide, mainly in Latin American countries, where it is considered endemic and reaches about 10,000 deaths per year.^1^ The highest morbidity and mortality of patients are associated with the symptomatic chronic phase (cardiac, digestive, or mixed clinical forms of the disease), which affects about 30% of patients. On the other hand, 70% progress to the indeterminate form, which is asymptomatic.^2^ ^,^ ^3^ The drug recommended for treating of CD in Brazil is Benznidazole (BZ). However, the results obtained from treatment vary according to the stage of the disease, clinical forms, dose, age, geographic origin, and the different susceptibility of the *T. cruzi* strains to drugs.^4^

The immunological mechanisms related to the clinical evolution of CD are not yet fully elucidated. However, it is believed that the association of the genetic background, the host’s immune response, and factors associated with the parasite are important in the patient’s clinical evolution.^5^ *T. cruzi* is highlighted as a versatile protozoan with all the necessary machinery to evade the immune response and persist for years without apparent symptoms.^6^ In this context, adipose tissue (AT) has emerged as a potential reservoir of infection for *T. cruzi*.^7^ ^,^ ^8^ ^,^ ^9^ ^,^ ^10^

The AT is an endocrine organ, as it can assume different phenotypes according to the stimulus applied. It acts both in metabolic homeostasis and in the immunoregulation of bioactive factors secreted by adipocytes.^11^ AT is divided into white (WAT), which is the primary site of energy storage through the storage of triglycerides, and the production and secretion of bioactive factors known as adipokines; brown (BAT) being associated with thermogenesis mechanisms. More recently, beige AT, which is an intermediate species between WAT and BAT that also has thermogenic properties.^12^ ^,^ ^13^

AT infection by *T. cruzi* was described for the first time by Shoemaker et al.^14^ in the BAT. However, only from the study by Combs et al.,^15^ the metabolic consequences of a *T. cruzi* infection in the AT were observed. Nagajyothi et al.^16^ showed that after infecting mice with the Brazil strain of *T. cruzi* for 15 days, both BAT and WAT showed high parasitaemia and macrophage influx in mice, being a target of the parasite in the early stages of the disease. On the other hand, Ferreira et al.^7^ detected the presence of parasite kDNA in subcutaneous AT samples discarded in the pacemaker placement procedure of individuals with CD, demonstrating the persistence of *T. cruzi* in the chronic phase of the disease.

Since AT acts as a reservoir of infection, the effectiveness of treatment with BZ may be controversial, as this tissue could act as a barrier to the drug’s action. Furthermore, the success of the treatment depends not only on the administration of the drug, but also on the synergistic effect of the drug and the host’s immune response.^4^ ^,^ ^6^ In this context, our study investigated the immunomodulation caused by BZ treatment in human AT infected with *T. cruzi*.

## MATERIALS AND METHODS

*Parasites* - Trypomastigote forms of the Y strain of *T. cruzi* were kept frozen in liquid nitrogen, were thawed and used for infection of Vero cells cultured in RPMI 1640 medium (Sigma^TM^) supplemented with 10% foetal bovine serum (FBS) (GIBCO^®^) and 1% penicillin /streptomycin (Lonza) (complete medium) for 24 h in a CO_2_ oven at 37ºC. At the end of the incubation, the parasites that did not infect the cells were removed. Then, a complete RPMI 1640 medium (Sigma^TM^) was added, and the cultures were incubated for five to eight days. Vero cells were observed daily under an inverted microscope. After the rupture of the Vero cells, the free trypomastigotes in the culture medium were collected, centrifuged (2555 x g for 10 minutes at 20ºC), and the precipitate was counted with Trypan Blue (Sigma^TM^) to observe the cell viability of the trypomastigotes that were used in culture.

*Cultivation of human adipose tissue-derived stem cells (ADSC)* - ADSC (PT-5006, LONZA^TM^), obtained through liposuction procedures, after thawing, were cultivated in 75 cm³ culture flasks in Dulbecco’s modified eagle medium (DMEM) supplemented culture medium with 20% FBS and 1% penicillin-streptomycin, and when they reached confluence, they were expanded with 2% trypsin/EDTA solution (GIBCO^TM^). All experiments with ADSC were performed between the 3rd and 6th cell passage.

*Adipogenic differentiation* - When they reached confluence, the ADSC were expanded in 2% trypsin/EDTA solution (GIBCO^TM^) and plated at a concentration of 5 x 10^5^ cells/well in individual culture plates of approximately 9.60 cm² in area. When they reached about 90% confluence (48 h post-plating), the differentiation wells were cultured with DMEM supplemented with 10% FBS and 1% gentamicin-amphotericin (PT-8205, LONZA^TM^) with the adipogenic inducers (recombinant human insulin, dexamethasone, 3-isobutyl-methyl-xanthine and indomethacin), according to the recommendations of the adipogenic differentiation kit (PT-9502, LONZA^TM^), for 12 days. The control group was cultured with DMEM supplemented with 10% FBS and 1% gentamicin-amphotericin. After the culture time recommended by the manufacturer, the wells were stained with the AdipoRed^TM^ reagent (PT-7009, LONZA^TM^), which uses the Nile Red dye to highlight the lipid vesicles. After adding AdipoRed^TM^ to the wells, the fluorescence of the lipid vesicles was visualised in the 485nm filter under 572nm emission, in the confocal microscope, according to the manufacturer’s recommendations.

**Fig. 1:**
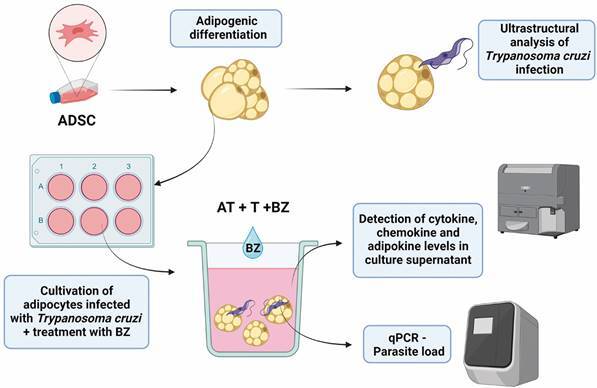
schematic representation of the study’s experimental design. Adipose-derived stem Cells (ADSC) were subjected to adipogenic differentiation. The differentiated adipocytes were infected with *Trypanosoma cruzi* and subjected to transmission electron microscopy. For immunomodulation, we cultured the differentiated adipocytes, infected them with *T. cruzi* and treated them with Benznidazole. After incubation, we removed the infected adipocytes to assess the parasite load and collected the culture supernatant to measure chemokines, cytokines and adipokines. Created with BioRender.com

**Fig. 2:**
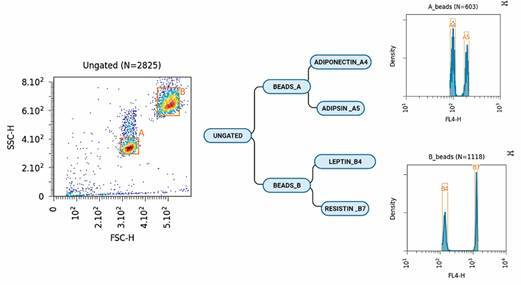
adipokines acquisition and analysis strategy. The adipokine population was selected using pseudocolour density (FSC) versus side scatter (SSC) plots. Eight hundred beads were acquired from window A (Beads A - Adiponectin and Adipsin) and 800 from window B (Beads B - Leptin and Resistin). The beads were classified using the FL4 channel. After selecting the window of interest (A) and (B), the adipokines were analysed by obtaining two-dimensional graphs of the point fluorescence distribution using LEGENDplex™ Data Analysis (Biolegend^®^).

**Fig. 3:**
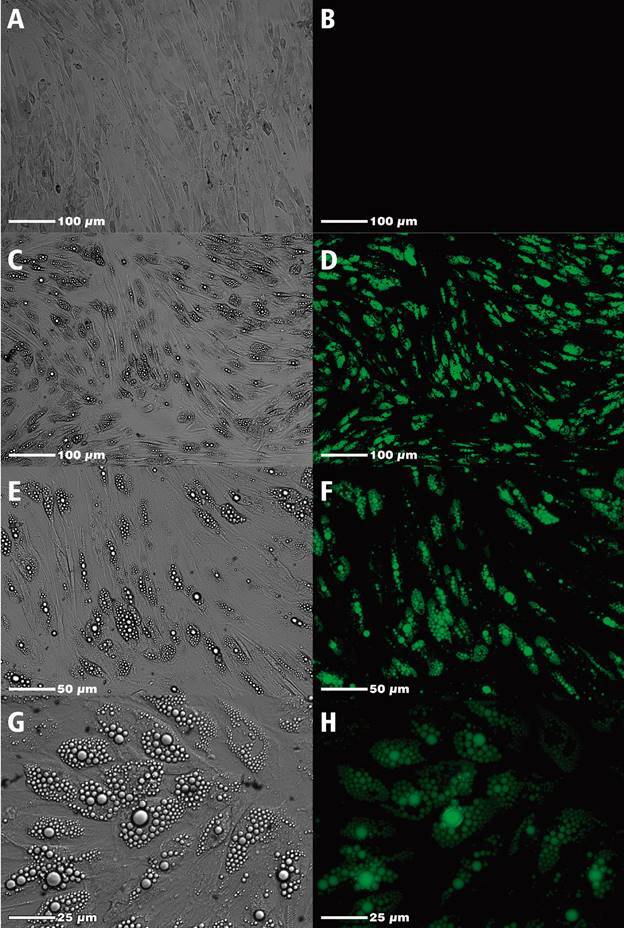
adipogenic differentiation of human Adipose-Derived Stem Cells (ADSC). A and B - ADSC cultured with basal medium (culture medium without adipogenic inducers); C-H - ADSC cultured with adipogenic differentiation medium. Green-stained lipid droplets represent fluorescence when stained in AdipoRed and viewed under a confocal microscope.

**Fig. 4:**
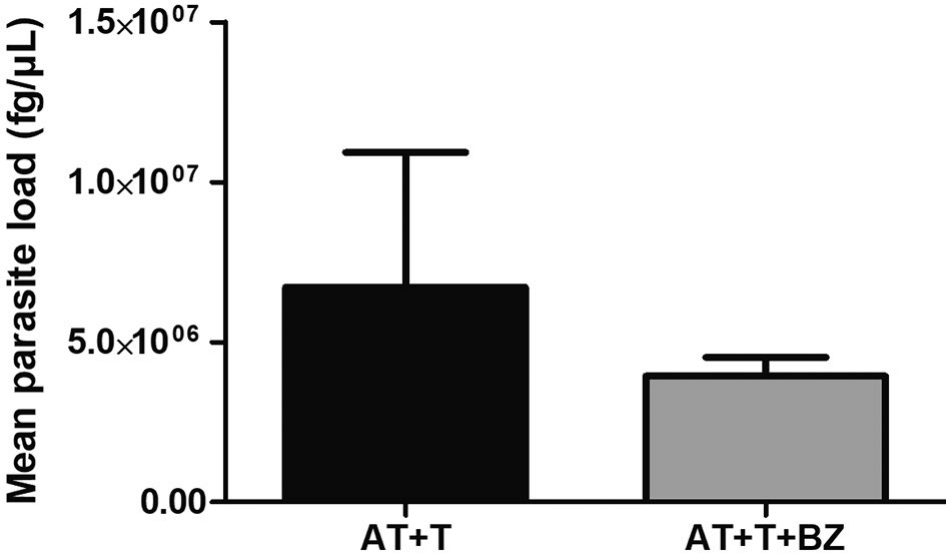
quantification of the parasite load of the culture of adipose tissue (AT) infected with *Trypanosoma cruzi* (T) and treated with Benznidazole (BZ) (n = 4). Data are expressed as averages ± standard deviations. Statistical analyses were performed using the Wilcoxon test to assess the differences between the groups; p-values < 0.05 were considered significant.

*Culture of T. cruzi-infected adipocytes and treatment with BZ* - After adipogenic differentiation, adipocytes were infected with trypomastigotes of the Y strain, derived from the culture of Vero cells to obtain trypomastigotes. Infection was carried out for 3 h, at a ratio of 5:1 parasites per cell, according to the protocol described by Nagajyothi et al.^17^ Parasites that failed to internalise were removed by washing and a DMEM medium supplemented with 10% FBS and 1% gentamicin-amphotericin (basal) was added. After 48 h of infection, treatment with BZ at a concentration of 1 µg/mL was added, according to the dose established for *in vitro* assays in the study by Romanha et al.^18^ Four independent experiments were carried out to compare the results and the cultivation time was 72 h after treatment with BZ. After the end of the culture, the supernatants were collected, and the cells were removed to quantify the parasite load (Fig. 1). The incubation time was estimated after time-response kinetics [Supplementary data (Figs 1-4)].

*Evaluation of the parasite load of T. cruzi-infected adipocytes in culture* - After the incubation time, the cells from each group were removed from the culture plates by washing with chilled phosphate-buffered saline (PBS) and promptly deposited in polypropylene tubes (BD Falcon^TM^ - 15 mL) for washing with PBS (500 x g, 10 min, brake 1). The final pellet was then transferred to microtubes, and DNA extraction was carried out using a commercial kit (QIAamp DNA Mini Kit - QIAGEN) according to the manufacturer’s recommendations. By combining primers (TcSAT1- F 5′AAATTCCTCCAAGCAGCGGA3′; TcSAT2 - R 5′ ATGAATGGCGGGAGTCAGAG3′), *T. cruzi* SAT-DNA detection systems were created and *T. cruzi* genomic DNA (strain Y) was used as a standard curve to quantify the parasite load. The experiments were conducted using the QuantStudio 5 real-time polymerase chain reaction (RT-PCR) System (Thermo Fisher Scientific) using the TcSAT-IAM system.^19^ The amplification of the human G3PD gene was used as a quality control for the samples used in the molecular reactions [Supplementary data (Figs 1-4)]. Samples were tested in duplicate at all stages. The results were analysed, interpreted, and recorded using the QuantStudio Design and Analysis Software.

*Ultrastructural analysis* - *T. cruzi-*infected adipocytes were fixed overnight with 2.5% glutaraldehyde in 0.1M cacodylate buffer. After that, the samples were post-fixed in a solution containing 1% osmium tetroxide, 2 mM calcium chloride and 0.8% potassium ferricyanide in 0.1M cacodylate buffer. The samples were counterstained with 2.5% uranyl acetate, dehydrated in increasing acetone series and embedded in Embed-812 resin. Ultrathin sections of the samples were stained with 5% uranyl acetate and 1% lead citrate and then visualised with a FEI Tecnai Spirit transmission electron microscope operated at 120 kV.

*Mensuration of chemokines and cytokines in the culture supernatant* - Culture supernatants were collected for the measurement of chemokines (CXCL10/IP-10, CCL2/MCP-1, CXCL9/MIG, CCL5/RANTES and CXCL8/IL -8) and cytokines (IL-6, IL-10, TNF-α and IFN-γ) through CBA (cytometric bead array-BD Biosciences, USA), according to the manufacturer’s recommendations. The samples were read on the Flow Cytometry Technological Platform, located at the Núcleo de Plataformas Tecnológicas (NPT)/IAM/Fiocruz, through the FACScalibur flow cytometer (Becton Dickson Immunocytometry Systems), with the CellQuestPro software (Beckton Dickson) and analysed in the FCAP 3.1 software.

*Mensuration of adipokines in the culture supernatant* - After collecting the culture supernatants, we measured the adipokines (adiponectin, adipsin, leptin and resistin) using the LEGENDplex^TM^ Human Metabolic Panel 1 kit (Biolegend^®^), according to the manufacturer’s recommendations. The samples were read on the Flow Cytometry Technology Platform, located at the NPT/IAM/Fiocruz, using the FACScalibur flow cytometer (Becton Dickson Immunocytometry Systems), with the BD CellQuestPro software (Beckton Dickson) and analysed using the LEGENDplex™ Data Analysis software (Biolegend^®^). The adipokine population was selected using pseudocolour density (FSC) versus side scatter (SSC) plots. Eight hundred beads were acquired from window A (Beads A - Adiponectin and Adipsin) and 800 from window B (Beads B - Leptin and Resistin). The beads were classified using the FL4 channel. After selecting the window of interest (A) and (B), the adipokines were analysed by obtaining two-dimensional graphs of the point fluorescence distribution using LEGENDplex™ Data Analysis (Biolegend^®^) (Fig. 2).

*Statistical analysis* - It was performed using the PRISM 8.0 Windows^®^ software (USA), where the results were submitted for verification of data normality to the Shapiro-Wilk test. The Wilcoxon test was used to analyse the parasite load results after carrying out the normality test of the samples. For cytokines, chemokines, and adipokines we used the one-way analysis of variance (ANOVA) test with Tukey’s post-hoc and Friedman test with Dunn’s post-hoc to assess the differences between the groups. All conclusions were taken at the 5% significance level.

## RESULTS

After the induction for adipogenic differentiation, we observed that lipid vesicles were not observed in the control well, where differentiation was not induced (Fig. 3A-B). In the differentiation wells, the ADSC had numerous lipid vesicles of variable size; however, for the most part, multilocular droplets (Fig. 3C-H), demonstrating that adipogenic differentiation was successful.

**Fig. 5:**
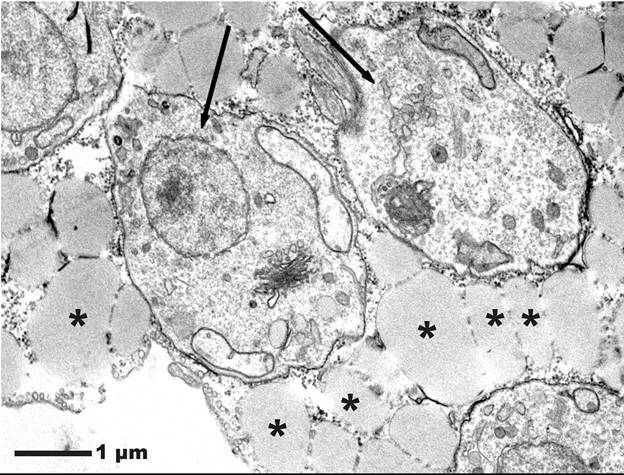
ultrastructure of *Trypanosoma cruzi*-infected differentiated adipose-derived stem cells (ADSC). Observe amastigote forms (arrows) between lipid droplets (asterisks) inside the adipose tissue (AT) cell.

In the evaluation of the average parasite load, we observed that the culture conditions infected with *T. cruzi* (AT+T and AT+T+BZ) showed considerably high parasite load (Fig. 4). As expected, the conditions AT and AT+BZ showed no detectable parasite load, so they were not graphically demonstrated. Furthermore, we observed that the AT+T+BZ (x̅: 3,9 x 10^6^) condition had a lower parasite load than the AT+T (x̅: 6,7 x 10^6^) condition (Fig. 4), although not statistically significant (p = 0.1667). Furthermore, in the ultrastructural evaluation, it was also possible to confirm the infection of the AT, through the visualisation of the amastigote forms of *T. cruzi* among the lipid droplets of the differentiated AT (Fig. 5).

**Fig. 6:**
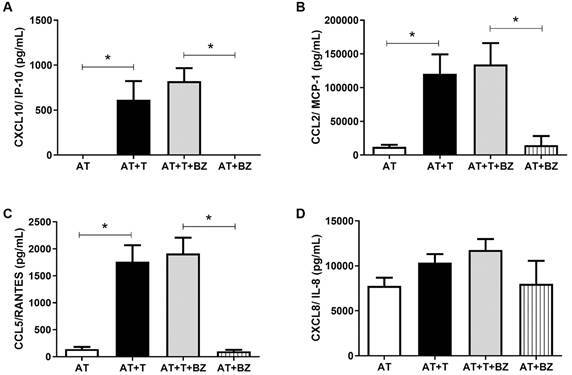
evaluation of the production of chemokines in the supernatant of the culture between adipose tissue (AT), *Trypanosoma cruzi* (T) and treatment with Benznidazole (BZ) (n = 4). Data is expressed as averages ± standard deviations. Statistical analyses were performed using the One-way analysis of variance (ANOVA) test with post hoc Tukey or Friedman test with post hoc Dunn’s to assess the differences between the groups; p-values < 0.05 were considered significant.

To verify the immunomodulation between AT infected by *T. cruzi* and treated with BZ, we performed culture and measurement of chemokines, cytokines and adipokines in the culture supernatant. Regarding the production of chemokines, it was demonstrated that *T. cruzi* infection (AT+T and AT+T+BZ) promotes a robust increase in CXCL10/IP-10, being statistically significant compared to AT (p = 0.0282) and AT+BZ (p = 0.0045) controls (Fig. 6A). The same phenomenon occurred for CCL2/MCP-1, compared to AT (p = 0.0110) and AT+BZ (p = 0.0076), and CCL5/RANTES compared to AT (0.0053) and AT+BZ (0.0029) (Fig. 6B-C). The results were not expressed graphically for the production of CXCL9/MIG because none of the evaluated culture conditions produced this chemokine. However, for CXCL8/IL-8, we observed that the production of this chemokine occurred consistently in all the culture conditions evaluated, with only a slight increase in the infected conditions (AT+T and AT+T+BZ) but no statistical difference (Fig. 6D).

**Fig. 7:**
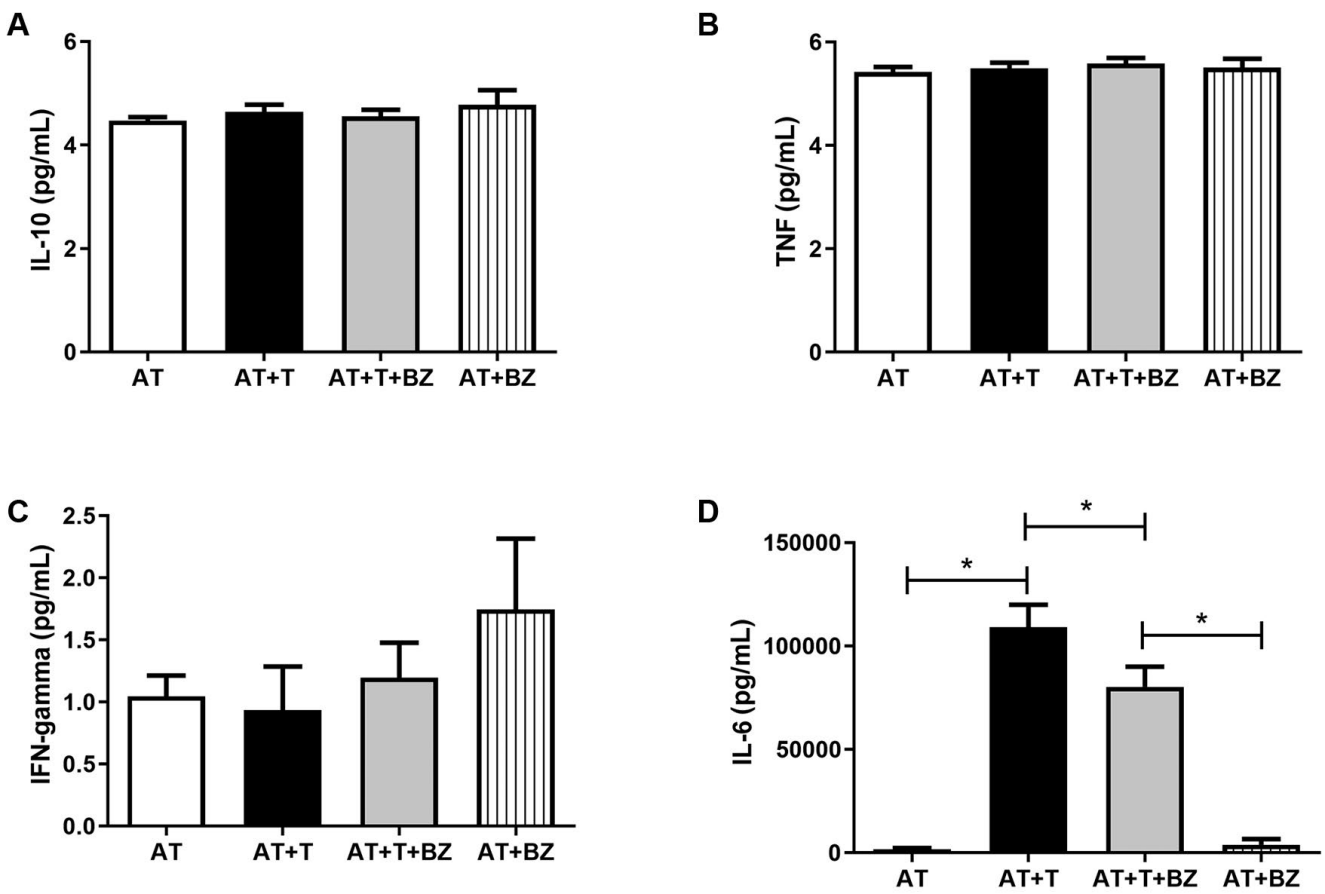
evaluation of the production of cytokines in the supernatant of the culture between adipose tissue (AT), *Trypanosoma cruzi* (T) and treatment with Benznidazole (BZ) (n = 4). Data is expressed as averages ± standard deviations. Statistical analyses were performed using the One-way analysis of variance (ANOVA) test with post hoc Tukey or Friedman test with post hoc Dunn’s to assess the differences between the groups; p-values < 0.05 were considered significant.

However, for cytokines, it was observed that IL-10 and TNF behave similarly under all culture conditions, regardless of BZ treatment (Fig. 7A-B). For the cytokine IFN-γ, basal production was also observed and similar between the analysed culture conditions, (Fig. 7C). Nevertheless, about the IL-6 cytokine, we observed discrepant results from the other cytokines measured, where AT+T and AT+T+BZ showed high levels of IL-6, being statistically significant to AT (p = 0.009) and AT+BZ (p = 0.0019). However, in the infected condition that was treated with BZ (AT+T+BZ), observed a decrease in IL-6 secretion compared to the infected condition (AT+T), which was statistically significant (p = 0.0352) (Fig. 7D).

**Fig. 8:**
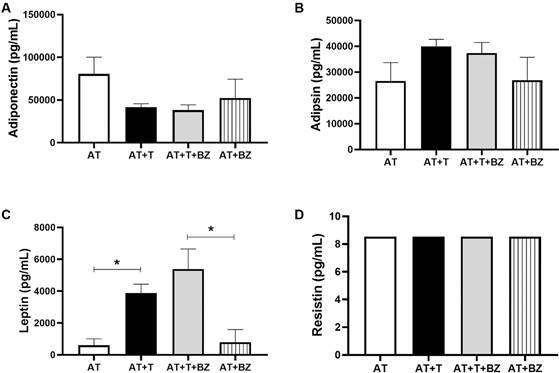
evaluation of the production of adipokines in the supernatant of the culture between adipose tissue (AT), *Trypanosoma cruzi* (T) and treatment with Benznidazole (BZ), (n = 4). Data is expressed as averages ± standard deviations. Statistical analyses were performed using the One-way analysis of variance (ANOVA) test with post hoc Tukey or Friedman test with post hoc Dunn’s to assess the differences between the groups, p-values < 0.05 were considered significant.

Concerning adipokines, we observed widespread secretion of adiponectin and adipsin in all culture conditions but with no statistical difference (Fig. 8A-B). However, leptin secretion was high in the infected culture conditions (AT+T and AT+T+BZ) when compared to AT and AT+BZ (p = 0.0013 and p = 0.0370) (Fig. 8C). Resistin secretion remained at baseline levels in all the groups evaluated (Fig. 8D).

## DISCUSSION

To verify the immunomodulation of infected and BZ-treated AT, we carried out the adipogenic differentiation of human ADSCs, where it was possible to observe the presence of small multilocular lipid droplets in the cytoplasm of the adipocytes. Depending on the maturation stage of AT, progenitor cells such as ADSC, pre-adipocytes in an intermediate stage or mature adipocytes can be found. In addition to adipocytes, AT is also composed of fibroblasts, endothelial cells, and smooth and skeletal muscle cells, which at times of inflammation are infiltrated by macrophages and leukocytes.^20^

In the study by Rashnonejad et al.^21^ the adipogenic differentiation of mesenchymal stem cells collected from liposuction procedures was performed in WAT and BAT, through the adipogenic inducers recommended by the manufacturer for each cell type. Then, it was observed that the differentiated cells had discrepant morphologies in terms of size of the lipid vesicles, BAT presented small and multilocular vesicles compared to WAT. Other studies have also observed the morphological discrepancy between WAT and BAT.^11^ Given this, our findings in adipogenic differentiation are morphologically like BAT or beige AT. However, specific markers are needed for more accurate classification.

In this context, such a heterogeneous and metabolically active microenvironment has become a promising target for bacteria,^22^ viruses^23^ ^,^ ^24^ and parasites,^25^ including *T. cruzi*.^17^ In ultrastructural analyses, amastigotes of *T. cruzi* have already been seen among lipid vesicles from murine cells.^15^ ^,^ ^17^ However, in our study, amastigote forms of *T. cruzi* were observed between the lipid vesicles of the AT differentiated from human ADSC, strengthening the hypothesis that *T. cruzi* can use AT as a reservoir in both mice and humans. Nevertheless, in vivo research that explores the parasite in a possible stage of dormancy/latency could be interesting to strengthen this theory.

Regarding the quantification of the parasite load, it was demonstrated through our results that *T. cruzi* can infect human AT *in vitro*, as well as multiply within these cells, according to the infected culture conditions. In agreement with our study, Ferreira et al.^7^ detected parasite kDNA in AT samples discarded in the pacemaker placement procedure of individuals with chronic CD, also proving AT infection in humans.

On the other hand, in the treatment with BZ, we verified a decrease in the parasite load in the AT+T+BZ condition, although there was no statistically significant difference between the AT+T condition. However, several studies have already shown that BZ has a beneficial effect, even in the chronic phase, by reducing the infection and/or promoting immunomodulatory effects *in vivo*, *in vitro* and *ex vivo*.^26^ ^,^ ^27^ ^,^ ^28^ Nevertheless, the drug has yet to be studied in human fat cells despite being a potential reservoir of infection for *T. cruzi*.

Regarding chemokines, we observed that *T. cruzi* infection promotes a robust increase in chemokines in infected AT. As we used differentiated AT, CXCL9/MIG was not produced under any of the conditions evaluated because its primary source was macrophages.^29^

In agreement with our study, Nagajyothi et al.^17^ also found increased chemokines CCL2, CCL5 and CXCL10 expression in murine fibroblasts, differentiated into adipocytes and infected by *T. cruzi*. In another study, it was also shown that the expression of CCL2, CCL5, CXCL8 and CXCL10 in CD-1 mice infected with *T. cruzi* was higher in BAT than in WAT, suggesting that such chemokines may have different results depending on the type of AT that was infected.^8^ ^,^ ^16^ Therefore, chemokine production can be affected by tissue type, metabolic diseases, and inflammation, among other disorders.^30^ Therefore, similar results in the production of chemokines were observed in both murine AT^16^ and human adipose cells in our study.

Treatment with BZ did not promote immunomodulation statistically different from that observed in AT+T conditions regarding chemokines. In the study by Albareda et al.,^31^ it was observed that the secretion of CCL2/MCP-1 in the culture supernatant of peripheral blood mononuclear cells (PBMC) from children infected with *T. cruzi* and treated with BZ/Nifurtimox decreased after six to 12 months of treatment. Even in IFN-γ-producing cells, there was an initial increase in the chemokine, which only decreased again after 24 months of treatment. Therefore, we believe that the time and dose of treatment may be a determining factor in immunomodulation, especially in the case of a biologically different cell.

Regarding cytokines, we highlight IL-6, which showed a robust increase in conditions infected by *T. cruzi*, compared to controls. IL-6 is a pro-inflammatory cytokine associated with the most severe clinical forms of CD. High levels of IL-6 are associated with greater severity in patients with CD and in murine infection models.^32^ In AT, in agreement with our results, Cabalén et al.^33^ found that infected mice on a high-fat diet increased IL-6 and MCP-1 in their plasma. Similarly, González et al.^34^ observed that IL-6 secretion remained high in infected fat cells, both from the 3T3-L1 strain and AT derived from infected mice.

In contrast, our findings showed that the AT+T+BZ condition decreased IL-6 compared to AT+T. Although the immunomodulatory effect of BZ has not yet been investigated in AT, in the study by Cevey et al.,^35^ it was shown that mice infected with *T. cruzi* when treated with low doses of BZ show a decrease in IL-6 and IL1-β. Therefore, if the administration of BZ promotes this decrease in IL-6, our data corroborate the hypothesis that treatment with BZ reduces exacerbated inflammation, even when treated with AT.

Looking at the adipokines, we found that adiponectin is secreted robustly in all culture conditions. This adipokine has anti-inflammatory properties and negatively regulates macrophage activation. A decrease in adiponectin is associated with obesity, insulin resistance and type 2 diabetes in rodents and humans.^36^ In *T. cruzi* infection, studies have already reported a decrease in adiponectin secretion.^15^ ^,^ ^16^ ^,^ ^34^

However, leptin levels in infected culture conditions are higher than in controls. Leptin is a pro-inflammatory adipokine produced mainly by the AT in proportion to body fat deposits. It regulates food intake, neuroendocrine function, reproduction, angiogenesis and blood pressure.^37^ In addition to our findings, Wueest et al.^38^ observed that IL-6 secretion induces the release of leptin by adipocytes. In another study, González et al.^39^ found that leptin secretion was higher in chagasic patients than in healthy individuals. However, Fernandes et al.^40^ observed that leptin levels vary according to the clinical forms of CD and suggested that its decrease is associated with heart failure. Therefore, although leptin is a pro-inflammatory adipokine, we believe that immunometabolic balance is necessary to control the inflammation caused by the parasite.

In view of the findings, we believe that treatment with BZ has a beneficial effect on infected AT, as it can reduce IL-6. Therefore, the results of the present study demonstrate that the drug can reduce inflammation and the parasitic load on adipocytes, in which *T. cruzi* uses AT as a reservoir of infection to evade the immune response.
