# Supplementary material for: Benznidazole treatment decreases IL-6 levels in Trypanosoma cruzi-infected human adipocytes differentiated from adipose tissue-derived stem cells
Source: Mem Inst Oswaldo Cruz. 2023 Oct 23;118:e220295. doi: 10.1590/0074-02760220295 (PMC10599316; doi:10.1590/0074-02760220295)
Supplement: Supplementary file 1 [file 1678-8060-mioc-118-e220295-s.pdf]

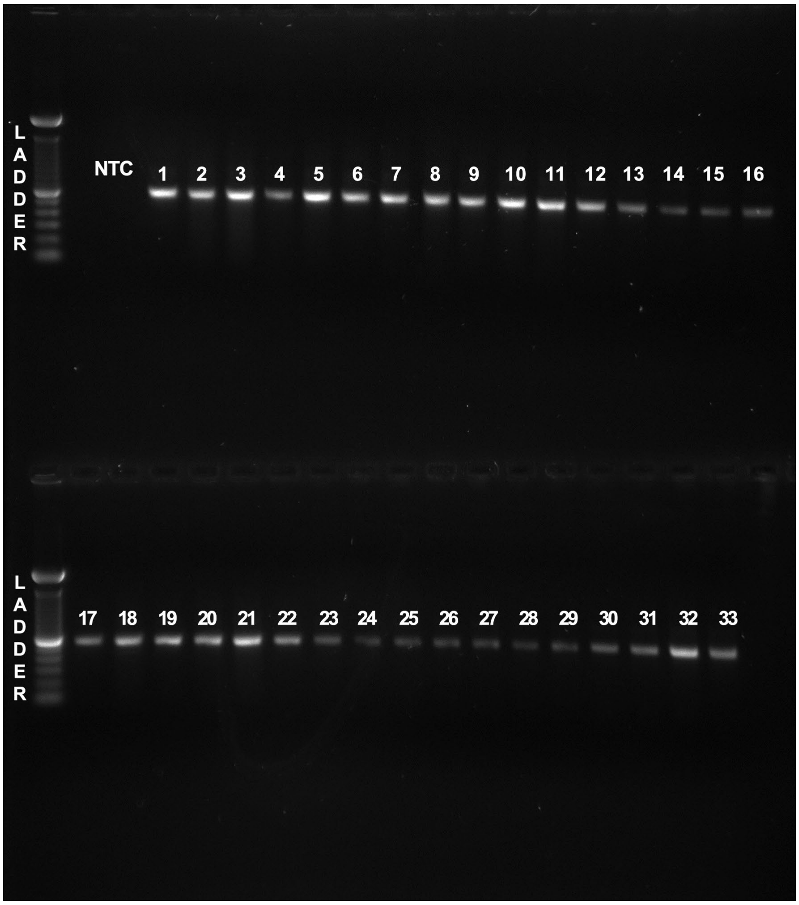

Fig. 1: amplification of the human G3PD gene for the extracted samples in the culture standardisation between ADSC and AT infected with *Trypanosoma cruzi* and treated with Benznidazole. Caption: NTC - Negative control; 1 - Positive control; 2 - ADSC 24 h; 3 - ADSC+T 24 h; 4 - ADSC+T+BZ 24 h; 5 - ADSC 48 h; 6 - ADSC+T 48 h; 7 - ADSC+T+BZ 48 h; 8 - ADSC 72 h; 9 - ADSC+T 72 h; 10 - ADSC+T+BZ 72 h; 11 - ADSC 96 h; 12 - ADSC+T 96 h; 13 - ADSC+T+BZ 96 h; 14 - AT 24 h; 15 – AT+T 24 h; 16 - AT+T+BZ 24 h; 17 - AT DM+T 24 h; 18 - AT DM+T+BZ 24 h; 19 - AT 48 h; 20 - AT+T 48 h; 21 - AT+T+BZ 48 h; 22 - AT DM+T 48 h; 23 - AT DM+T+BZ 48 h; 24 - AT 72 h; 25 - AT+T 72 h; 26 - AT+T+BZ 72 h; 27 - AT DM+T 72 h; 28 - AT DM+T+BZ 72 h; 29 - AT 96 h; 30 - AT+T 96 h; 31 - AT+T+BZ 96 h; 32 - AT DM+T 96 h; 33 - AT DM+T+BZ 96 h. ADSC - Adipose Derived Stem Cells; AT - Adipose tissue; DM - Differentiation medium; T - *Trypanosoma cruzi*; BZ - Benznidazole.

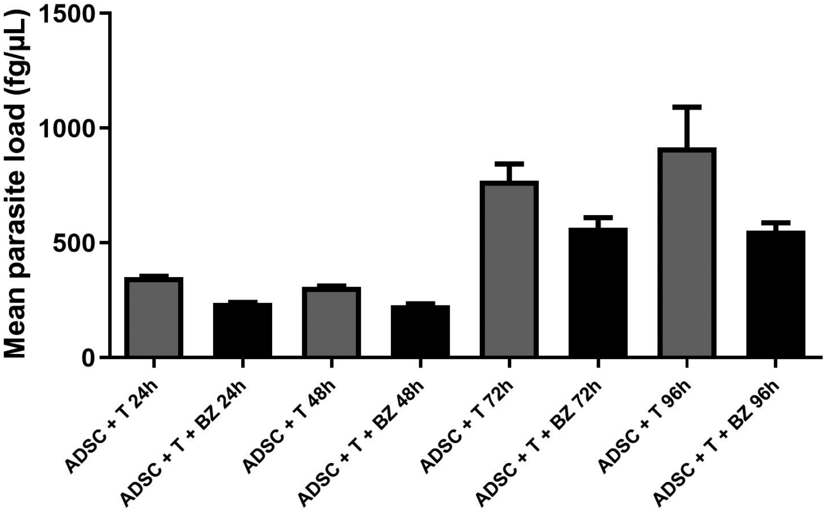

Fig. 2: quantification of the parasite load of standardised culture of ADSC infected with *Trypanosoma cruzi* and treated with Benznidazole. Caption: ADSC - Adipose-Derived Stem Cells; AT - Adipose tissue; DM - Differentiation medium; T - *Trypanosoma cruzi*; BZ - Benznidazole.

CC BY

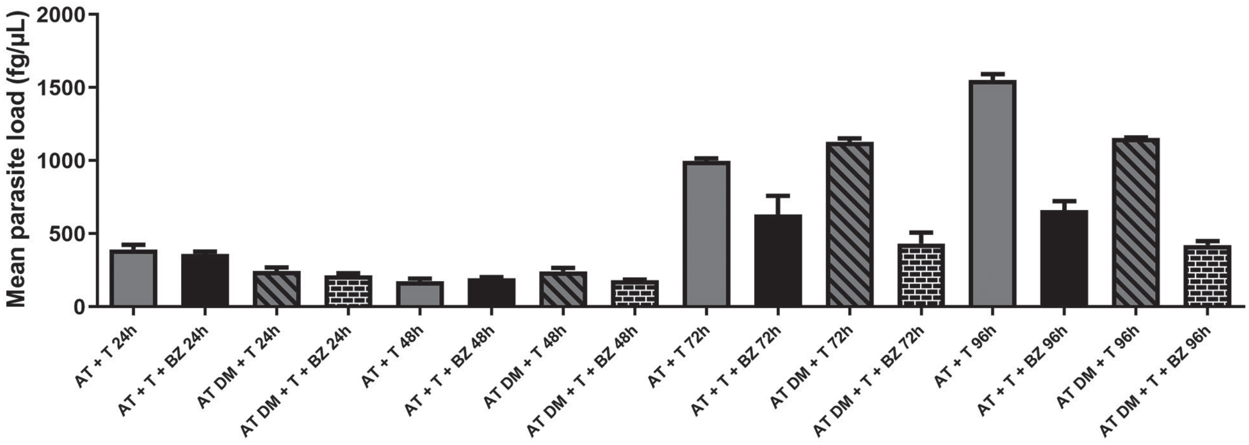

Fig. 3: quantification of the parasite load of standardised culture of AT infected with *Trypanosoma cruzi* and treated with Benznidazole. Caption: AT - Adipose tissue; DM - Differentiation medium; T - *Trypanosoma cruzi*; BZ - Benznidazole. Comment: As this was a standardisation, infection in differentiation medium (AT DM) and basal medium (AT) was assessed. Subsequently, we adopted the method that did not alter the immunomodulation results due to the inducers' effects.

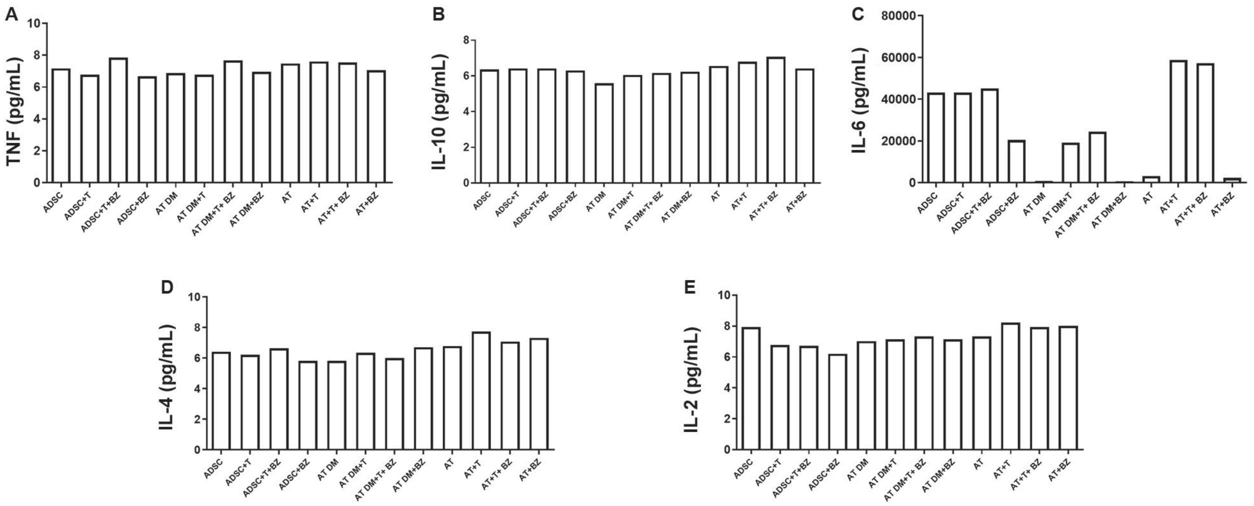

Fig. 4: measurement of cytokine levels in the culture supernatant of the standardised culture between ADSC and AT, infected with *Trypanosoma cruzi* and treated with Benznidazole, at 72 h. Caption: ADSC - Adipose-Derived Stem Cells; AT - Adipose tissue; DM - Differentiation medium; T - *Trypanosoma cruzi*; BZ - Benznidazole. Comment: As this was a standardisation, infection in differentiation medium (AT DM) and basal medium (AT) was assessed. Subsequently, we adopted the method that did not alter the immunomodulation results due to the inducers' effects. Looking at the parasite load, we realised that 72 h might be the most appropriate time and measured the cytokines.
